# Instruments of Choice for Assessment and Monitoring Diabetic Foot: A Systematic Review

**DOI:** 10.3390/jcm9020602

**Published:** 2020-02-24

**Authors:** Raúl Fernández-Torres, María Ruiz-Muñoz, Alberto J. Pérez-Panero, Jerónimo García-Romero, Manuel Gónzalez-Sánchez

**Affiliations:** 1 Department of Nursing and Podiatry, Arquitecto Francisco Peñalosa, s/n, Ampliación campus de Teatinos, University of Málaga, 29071 Málaga, Spain; raulft.95@gmail.com (R.F.-T.); albertoj2p@hotmail.com (A.J.P.-P.); 2 Medical School of the Physical Education and Sports, C/ Jiménez Fraud 10, Edificio López de Peñalver, University of Málaga, 29010 Málaga, Spain; jeronimo@uma.es; 3 Department of Physiotherapy, Arquitecto Francisco Peñalosa, s/n, Ampliación campus de Teatinos, University of Málaga, 29071 Málaga, Spain; mgsa23@uma.es

**Keywords:** diabetes, diabetes complications, foot, ankle, outcome measures, tools, evidence, review

## Abstract

Diabetic foot is the most frequent disorder among the chronic complications of diabetes, happening in 25% of patients. Objective clinical outcome measures are tests or clinical instruments that provide objective values for result measurement. The aim of this study was to carry out a systematic review of specific objective clinical outcome measures focused on the assessment and monitoring of diabetic foot disorders. The databases used were PubMed, CINAHL, Scopus, PEDro, Cochrane, SciELO and EMBASE. Search terms used were foot, ankle, diabet*, diabetic foot, assessment, tools, instruments, objective outcome measures, valid*, reliab*. Because of the current published evidence, diabetic neuropathy assessment via sudomotor analysis, cardiovascular autonomic neuropathy and peripheral vascular disease detection by non-invasive electronic devices, wound 3D dimensional measurement, hyperspectral imaging for ulcer prediction and the probe-to-bone test for osteomyelitis diagnosis were highlighted in this study.

## 1. Introduction

Diabetes is one of the most common diseases and its incidence is growing fast, as seen by the exponential increase in global prevalence over the last 30 years [1]. Its incidence is predicted to continue rising from the current 5.1% to 7.7% in 2030 [2] and is expected to affect 642 million people in 2040 [3].

Diabetic foot is the most frequent condition among the chronic complications of diabetes, occurring in 25% of patients [4]. It is also one of the most expensive [5], with 20–40% of resources used in diabetes destined for foot problems [6]. Furthermore, it is the main cause of hospitalization and amputation in diabetic patients [5], to the extent that one limb is amputated every 30 s [2]. The most common risk factors are neuropathy (86% of cases), peripheral arterial disease (49% of cases), trauma and foot deformities [2].

The best strategy for prevention and management of diabetic foot involves adequate control of diabetes, complete foot assessment and healthcare based on prevention and education with the support of a multidisciplinary team [7].

There are two options for patient monitoring and assessment: objective clinical outcome measures (OCOMs) [8] and patient-reported outcome measures (PROMs) [9]. OCOMs and PROMs help to normalize results, minimize errors and improve the understanding of results by patients and clinicians [8]. OCOMs are tests or clinical instruments that provide objective values for result measurement with a degree of reliability and validity [8].

Although the lower limbs are the area most affected by diabetes and are exposed to severe complications, to our knowledge, there are no systematic reviews regarding OCOMs in diabetic foot disorders.

The main objective of this manuscript was to carry out a systematic review of specific OCOMs focused on the assessment and monitoring of diabetic foot disorders. In addition, two other objectives of this work were to analyze the psychometric properties of OCOMs and, accordingly, identify the instruments of choice that are of the highest quality.

## 2. Material and Methods

### 2.1. Protocol and Registration

This systematic review was carried out according to the general guidelines and recommendations made by the Preferred Reporting Items for Systematic Reviews and Meta-Analyses (PRISMA) [10] and was registered in the PROSPERO database (CRD no.: 42019118202).

### 2.2. Eligibility Criteria

The study population consisted of patients with diabetic foot disorders, regardless of age or type of diabetes. All studies, including valid OCOMs for diabetic foot assessment and monitoring, regardless of the type of intervention, were accepted. Documents published up to 30 March 2019 were included. Documents that were not published in English, Spanish, French, Italian or Portuguese were excluded. We excluded studies that regarded OCOMs without valid and reliable data or those that did not provide any of the psychometric properties of the Consensus-Based Standards for the Selection of Health Measurement Instruments (COSMIN) criteria [11].

### 2.3. Sources and Search

The databases used were PubMed, CINAHL, Scopus, PEDro, Cochrane, SciELO and EMBASE. The following search terms were used, along with ‘OR’ and ‘AND’ terms: foot, ankle, diabet*, diabetic foot, assessment, tools, instruments, objective clinical measures, valid*, reliab*.

According to each database, the following search strategy was used: (((foot OR ankle) AND (diabet*)) OR (diabetic foot)) AND ((assessment) OR (tools) OR (instruments) OR (objective clinical measures) OR (valid*) OR (reliab*)).

### 2.4. Study Selection

Three review authors independently participated in each stage of the study selection. First, they screened by titles and abstracts of the references identified through the search strategy. Full reports of all potentially relevant documents were then assessed for eligibility based on the eligibility criteria of this review. Differences of judgement were settled through discussion to achieve a consensus.

### 2.5. Data Extraction and Synthesis of Results

To facilitate understanding of the results, the outcome variables were classified into three categories, according to diagnostic purpose: variables related to diabetic neuropathy, peripheral vascular disease (PAD) and diabetic ulcer characteristics.

The methodological quality of the studies, showing the properties of the outcome measures, was rated on a four-point scale according to the COSMIN checklist [11]. This checklist was used to evaluate whether a study with subjective measurement tools meets the standards of good methodological quality. However, as this study was aimed at objective instruments, data extraction was adapted according to the following calculated properties: sensitivity, specificity, positive predictive value (PPV), negative predictive value (NPV), positive likelihood ratio (LR+), negative likelihood ratio (LR), area under the receiver operator characteristic curve (AUC-ROC), gold standard, agreement with gold standard, inter- and intra-rater reliability. Other results taken to help in understanding each study were the variables, OCOM nomenclature, type of diabetes and number of patients.

## 3. Results

**Figure 1:**
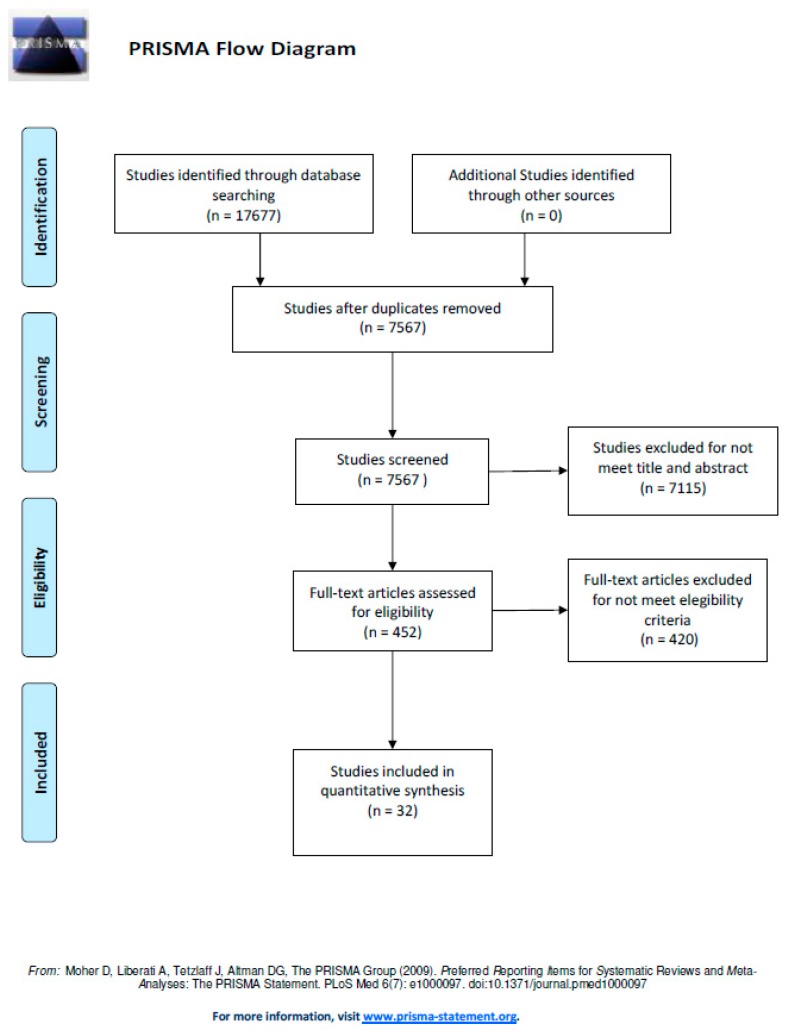
PRISMA Flow Diagram.

The flow diagram (Figure 1) summarize the study selection processes, including reasons for exclusions, at each stage for the studies included in this review [10].

After extracting the data provided by the studies included in this review, the variables were divided into three groups according to diabetic complications: neuropathy, PAD and ulcer-related characteristics.

Table 1 shows the variables related to diabetic neuropathy and the OCOMs validated for their assessment: 13 variables and 18 OCOMs were included in this category. The majority of the variables were related to peripheral neuropathy. Variables regarding the autonomic and proximal components of neuropathy are provided at the end of the table.

Table 2 shows the variables related to PAD and the OCOMs validated for their assessment: three variables and four OCOMs were included in this category.

Table 3 shows the variables related to ulcer characteristics and the OCOMs validated for their assessment: nine variables and 12 OCOMs were included in this category.

## 4. Discussion

The aims of the present study are to carry out a systematic review of the OCOMs focused on diabetic foot in order to analyze validated tools for diabetic foot assessment and evaluate the psychometric properties of the diabetic foot assessment tools. Our results show 35 OCOMs, measuring 26 outcome variables classified into three categories: variables related to diabetic neuropathy, PAD and diabetic ulcer characteristics. These aims were achieved in the study.

### 4.1. Psychometric Properties Calculated in OCOMs

Sensitivity and specificity were the most often calculated psychometric properties, knowing their values for 26 OCOMs (both calculated in all cases). These are the main psychometric properties for assessing the ability to detect true positives and true negatives, therefore they are essential in OCOM validation studies [41].

The positive predictive value (PPV) and negative predictive value (NPV) were calculated for 19 OCOMs, the positive likelihood ratio (LR+) for 12 OCOMs and the negative likelihood ratio (LR−) for 11 OCOMs. The calculation of 2 × 2 contingency tables, sensitivity and specificity was done prior to obtaining these four psychometric properties [42]. PPV and NPV reflect the impact of pathology prevalence in the validity property [43]. LR+ and LR− are important in terms of the likelihood of an OCOM to detect true negatives and true positives [44].

Inter-rater and intra-rater reliability were calculated for six and ten OCOMs, respectively. These two psychometric properties are essential when an OCOM shows variability in the results, either due to variability of the OCOM itself or the intervention required by the examiner.

### 4.2. Variables and OCOMs for Assessment of Diabetic Neuropathy

Fourteen variables measured by 19 OCOMs were found (see Table 1). These variables were classified into three subgroups, depending on the component of the diabetic neuropathy assessed: peripheral (distal polyneuropathy), proximal (amyotrophic or motor) and autonomic [45].

### 4.3. Variables and OCOMs for Assessment of the Peripheral Component of Diabetic Neuropathy

The outcome variable ‘assessment of peripheral neuropathy’ contains the most OCOMs for its measurement (ten): Neuropad, 10 g monofilament, Neurotip, 128 Hz tuning fork, Vibratip, NeurAppathy app, diabetic peripheral neuropathy (DPN) check, tactile circumferential discriminator (TDC), Sudoscan and the footboard (FB) system (Table 1).

Neuropad was the most sensitive (100%) and specific (100%) OCOM in this subgroup for the staging of peripheral neuropathy, depending on the color change threshold and according to the Michigan Neuropathy Screening Instrument (MNSI) [12].

In addition, Neuropad is valid for the measurement of two other variables related to peripheral neuropathy: small nerve fiber neuropathy (with sensitivity and specificity up to 83% and 80%, respectively) and large nerve fiber neuropathy (with sensitivity and specificity up to 83% and 64%, respectively) [22]. The former appears as an early manifestation of peripheral neuropathy closely linked to the autonomic component [14,46], which makes Neuropad a specific diagnostic tool valid for the assessment of both. In addition, it has shown excellent intra- and inter-rater reliability for peripheral neuropathy diagnosis (≥0.90).

NerveCheck measures more outcome variables (five) than any other OCOM in this subgroup, although its psychometric properties show variability depending on the selected variable [23,47]. It presents the lowest values of sensitivity (40%) and specificity (68%) for the assessment of neuropathic pain and its highest values for the assessment of large nerve fiber diabetic neuropathy (88% and 82%, respectively) (see Table 1). The external validity of NerveCheck and Neuropad has been calculated based on the density and length of the corneal nerve fiber, alleging its capacity to detect neuropathy earlier compared with any other method [14].

The footboard system was the OCOM with the highest sensitivity of 100%, PPV of 100% and NPV of 93% in this subgroup, although this validity depends on the variant of the instrument: for example, the 3 mm variant has 100% sensitivity but 9% specificity, whereas the 1 mm variant has 63% sensitivity but 90% specificity [18]. This range of psychometric properties, added to the lack of literature on this OCOM, suggests the need for further studies.

The 10 g monofilament and the 128 Hz tuning fork, in this order, were the most frequently used OCOMs according to this review. In comparison to the same gold standard (neurothesiometer), the 10 g monofilament had a significantly higher degree of external validity than the tuning fork [13].

The tuning fork was more specific (90%) than the 10 g monofilament (83%), but the 10 g monofilament was more sensitive (84%) than the tuning fork (69%). In the leprosy population (which implies a distal neuropathy similar to diabetics), the 10 g monofilament had lower sensitivity (38%) and greater specificity (91%) compared to those values in diabetes mellitus [48].

A meta-analysis published in 2017 does not recommend the 10 g monofilament for the diagnosis of peripheral neuropathy because of its low sensitivity (53%) compared to gold standard ‘nerve conduction studies’ (NCS) [49]. However, according to the results of this review, the 10 g monofilament has greater sensitivity (84%) compared to the neurothesiometer, which is frequently used as a gold standard [13,23]. The neurothesiometer has a very significant correlation with NCS for the assessment of peripheral neuropathy [25], therefore, in the present review, the neurothesiometer was included as a gold standard for the calculation of external validity.

### 4.4. Variables and OCOMs for Assessment of the Proximal Component of Diabetic Neuropathy

The manifestation of proximal neuropathy in the foot causes muscle atrophy, which leads to functional imbalance, generating overload and potential ulceration in risk areas [50]. In this review, ultrasonography studies show evidence for the diagnosis of intrinsic foot muscle atrophy, with a good degree of correlation with magnetic resonance imaging (MRI) results (*r*^2^ = 0.71–0.77) [26]. Ultrasonography is a good alternative to MRI as it is a faster, more economical and more practical diagnostic test. Moreover, it allows an active and live study of intrinsic muscle function [51]. It is known that the size measurement of the intrinsic foot muscles by ultrasound has an excellent inter-observer reliability (ICC = 0.90–0.97) [52].

### 4.5. Variables and OCOMs for Assessment of the Autonomic Component of Diabetic Neuropathy

Regarding autonomic neuropathy (Table 1), Sudoscan and Neuropad were the only OCOMs validated for its assessment [17,32], showing similar sensitivity (82% and 85.6%, respectively) and specificity (75% and 76.1%, respectively) (Table 1).

As the autonomic component of neuropathy is not exclusive to diabetes, Neuropad and Sudoscan have both proved to be valid for use in the detection of other diseases, such as amyloid polyneuropathy, leprotic neuropathy and Parkinson’s disease.

Regarding familial amyloid polyneuropathy, both Neuropad and Sudoscan were valid for the detection of asymptomatic, moderate and severe staged patients [53]. Similar to diabetes, Sudoscan shows 67.44% sensitivity and 83.33% specificity for the diagnosis of autonomic neuropathy in Parkinson’s disease, therefore it could be useful in both conditions [54]. Neuropad is valid for assessment of the autonomic neuropathy component in leprosy, although it has lower psychometric properties for this disease (56% sensitivity and 61% specificity) [48].

### 4.6. Variables and OCOM for the Assessment of a Diabetic Autonomic Neuropathy (DAN)

Apocket-size instrument (Vagus^®^) was specifically designed to measure the analysis of cardiovascular autonomic neuropathy by measuring the heart rate variability (HRV) through performing three tests (the response to active standing ratio (30:15), the Valsalva maneuver and expiration-to-inspiration ratio (E:I)) specifically designed to evaluate the parasympathetic nervous system, which is usually more affected than the sympathetic nervous system in the case of DAN. The external validity of this instrument was calculated using the Varia Pulse TF3 as a gold standard. Pearson’s correlation rates between both instruments ranged from *r^2^*= 0.81 to *r^2^*= 0.98 [19]. In addition, Vagus^®^ presented inter-subject reliability that ranged from good to excellent, while the intrasubject was excellent (Table 1) [55,56].

### 4.7. Variables and OCOMs for Assessment of Peripheral Arterial Disease (PAD) in Diabetes

Four OCOMs were found for PAD assessment in diabetes (see Table 2). The Novametrix 800 monitor had the highest sensitivity (98%), PPV (91%) and NPV (80%) for measurement of oxygen transcutaneous pressure (TcPO2) but also the lowest specificity (44%) [29].

According to this study, the Ankle-Brachial Index (ABI) was the most widely used OCOM, although, in a previous validation study, it showed low sensitivity (45.16%) for the diagnosis of PAD in diabetes using a classic mercury sphygmomanometer and eco-Doppler [28]. However, another study that evaluated the validity of a hybrid sphygmomanometer (OMRON HEM-907) against a classical sphygmomanometer for calculation of the ABI in diabetic patients obtained 77.5% sensitivity and 98.2% specificity [57]. Therefore, these values support the use of the ABI based on psychometric properties.

The Toe-Brachial Index (TBI) has a higher sensitivity than the ABI if a classic sphygmomanometer and eco-Doppler are used (63.64% versus 45.16%); regarding the TBI, the intra-observer reliability of the finger blood pressure measurement is ICC = 0.80, whereas, for the ABI, these values were 0.62 for ankle pressure and 0.66 for brachial pressure [28]. However, according to another study [58], there are no differences between the TBI and the ABI for the diagnosis of PAD in diabetic subjects unless arterial calcification exists (ABI > 1.3), in which case TBI assessment is recommended.

The Novametrix 800 monitor measures TcPO2, which evaluates foot skin blood supply objectively based on its oxygenation, which is responsible for maintaining skin integrity [59]. Its sensitivity in diabetes is excellent (98%), much greater than that for the detection of PAD from other aetiologies [29].

TcPO2 has been proposed by some authors as a diagnostic variable of peripheral diabetic neuropathy due to its origin in microangiopathy [60] (see Table 1), although it has lower sensitivity (61.1%) compared to PAD evaluation [20].

The OMRON BP-203RPEIII shows high sensitivity and specificity (94.5% and 98.99%, respectively) for the calculation of the ABI [30] but, because it does not require examiner intervention, inter-observer reliability was not relevant.

### 4.8. Variables and OCOMs for Assessment of the Characteristics of Diabetic Ulcers

A total of 10 variables and 13 OCOMs were found. The OCOM with the highest sensitivity and specificity (100%) was the hyperspectral imaging device, depending on the percentages of oxyhaemoglobin and deoxyhaemoglobin taken as the cut-off values [37].

The variable measured by the highest number of OCOMs (five) was the ‘diagnosis of osteomyelitis’, for which the probe-to-bone test was the most sensitive (98.1%), however, it is important to mention that this instrument has a high interrater variability [61]. In this sense, the gold standard for the diagnosis of osteomyelitis continues to be bone biopsy [61]. Plain radiography, positron emission tomography (PET), MRI and leukocyte counting were other OCOMs used for the diagnosis of osteomyelitis. The OCOMs with the highest PPV (96%) and NPV (94%) in this subgroup were MRI and PET, respectively, but they require more time and resources than the probe-to-bone test [33,62]. LR+ and LR− have only been calculated for the probe-to-bone test, which gives more support for its use.

The photographic foot imaging device (PFID) proved valid for the measurement of most variables: ulcer infection, diagnosis of ulcer, diagnosis of hyperkeratosis and absence of signs of skin risk [63,64].

The 3D wound assessment monitor (3DWAM) provided the most complete statistical study, with excellent external validity (ICC = 0.997) and inter- and intra-rater reliability (ICC = 0.997 and 0.999, respectively); in addition, the validation study was also performed on surgical, traumatic and pressure wounds [65]. Another instrument that presents excellent reliability for measuring the surface of the ulcer is ImageJ [31], with an inter-rater value of ICC = 1 and intra-rater of ICC = 0.99. [31]

Plasma fibrinogen was a valid measure to assess ulcer severity [66], which provides an alternative to ulcer severity scales, thus solving the drawback of clinician subjectivity.

These results complement those published in a systematic review focused on the analysis of different strategies/instruments for measuring the area and volume of wounds [67]. Specifically, in this systematic review, six different methods were identified to assess the volume/area of wounds: simple ruler method, mathematical models, manual planimetry, digital planimetry, stereophotogrammetry and digital imaging. Each instrument has a series of positive features, such as ease of use (simple ruler method, mathematical models, manual planimetry, digital planimetry), good precision (mathematical models, manual planimetry, digital planimetry, stereophotogrammetry and digital imaging) or economy of use (simple ruler method, mathematical models) [67]. However, they also have some limits that must be taken into account when they are used, such as lack of precision especially on rounded surfaces (simple ruler methods), the possibility of contaminating the wound (planimetry) or the time it takes to be able to measure the area/volume of the wound (stereophotogrammetry and digital imaging). Not all of these tools have been used to analyze diabetic foot ulcers, although in those where it has been performed, it is in line with the systematic review previously mentioned, although some important psychometric characteristics, such as intra-interobserver reliability, have not been analyzed [32]. Perhaps future studies could be developed to analyze the reliability, accuracy and validity of some of these instruments for the assessment of diabetic foot ulcers.

### 4.9. Clinical Recommendations for OCOMs Evaluated in the Review

Given that diabetic neuropathy has several components (peripheral, autonomic and proximal), it seems a good strategy to examine each one independently to make more accurate recommendations [61].

The widespread use of the 10 g monofilament for the assessment of peripheral neuropathy may be due to its low economic cost and speed of use, in addition to its high psychometric properties. However, according to a meta-analysis published in 2016, its use was not recommended due to its low external validity and, hence, it would not be the OCOM of choice [49]. Other studies did not recommend its use in a type 1 diabetic population of childhood age due to its low inter-observer reliability [65]. On the other hand, it is important to consider monofilament as a valuable tool due to its predictive ability to identify the greater or lesser risk of ulcers in patients with diabetes [66].

Neuropad seems a good choice because it is used for the diagnosis of both peripheral and autonomic components of diabetic neuropathy in type 1 and 2 diabetes [27]. Furthermore, it allows the distinction between the type of nerve fibers affected in peripheral neuropathy (small or large) and has excellent inter- and intra-observer reliability [22].

Neuropad and Sudoscan were presented as good options for the diagnosis of diabetic autonomic neuropathy based on their psychometric properties. In addition, they are also valid for other pathologies involving autonomic neuropathy [48,53,54]. Neuropad is valid for type 1 and 2 diabetes, but Sudoscan has only been studied in type 2 diabetes.

No OCOMs have been validated for the diagnosis of proximal neuropathy, although ultrasonography can detect muscle atrophy of the foot because it has good external validity with MRI. Only one study recommending its use has been found. The absence of cut-off values for the diagnosis of muscle atrophy makes the role of the examiner important in its assessment.

Regarding PAD diagnosis in diabetic patients, the OMRON BP-203RPEIII for calculation of the ABI has shown the best psychometric properties. As there are no differences in the diagnosis of PAD between the ABI and the TBI [58], it was recommended to calculate the ABI first, because it was quicker; however, if its value exceeded 1.30 (presence of arterial calcification), then measurement of the TBI should subsequently be performed.

For assessment of ulcer-related variables, the probe-to-bone test for the diagnosis of osteomyelitis seems to be the most valid in clinical practice, notwithstanding its low economic and time costs [38]. The 3DWAM was a valid and reliable OCOM [33], potentially applicable for follow-up of ulcer progress according to its dimensions and healing times.

The PFID was valid for assessing several skin lesions [37,39] but its application is limited to telediagnosis as in situ assessments by healthcare professionals remain the gold standard.

According to the results, hyperspectral imaging was valid for the prediction of ulcer onset in healthy skin [37].

Owing to its presence in two out of three groups in this review (see Table 1 and Table 2), TcPO2 measurement seems interesting because it shows validity for variables related to the diagnosis of peripheral neuropathy and PAD. However, sensitivity for the detection of peripheral neuropathy was low (61.1%), so it would be a better choice to use other OCOMs for this purpose.

Although, in this systematic review, an analysis of the psychometric characteristics of the instruments for the assessment and follow-up of patients with diabetic foot has been carried out, it is important to take into account that there are other factors that can become much more decisive than the psychometric characteristics of the instruments. For example, the cost, both in the acquisition of the instrument and in its use, can be a limitation in the selection of the instrument. In addition, not all instruments are available in all countries of the world, so the accessibility of the instrumentation necessary to perform an evaluation of diabetic foot will determine the choice of the instrumentation that can be used in the assessment and follow-up of patients with diabetic foot.

### 4.10. Research Recommendations for OCOMs Evaluated in the Review

The design of the validation studies did not allow for comprehensive discussion of all the psychometric properties of the OCOMs analyzed, so it is recommended to overcome this with future studies that facilitate the choice of clinicians and researchers; in most studies, although the sensitivity and specificity have been calculated after carrying out 2 × 2 contingency tables, calculation of PPV, NPV, LR+ and LR− has been missed in these studies of validation and it would be helpful to calculate all the psychometric properties of the OCOMs in order to facilitate comparison between them and elaborate on their level of evidence.

Another important finding has been the lack of inter- and intra-rater reliability data in the OCOMs analyzed in the review. This seems essential in those OCOMs where the intervention and interpretation of an examiner are needed for measurement, as with the Neuropad or 10 g mono filament. The latter requires the intervention of a patient and examiner, and with its low inter-rater reliability in children with type 1 diabetes [33] it would be advisable to use other valid OCOMs for this specific population. Hence, the inter- and intra-rater reliability of the 10 g monofilament should be studied in all other target populations. Likewise, Neuropad provides qualitative results (color changes) that need to be interpreted by an examiner; however, no studies have been found to calculate its inter- and intra-rater reliability, so this is recommended for future studies. In some OCOMs, such as the OMRON BP-203RPEIII or Sudoscan, this reliability is not as necessary because there is no requirement for an examiner, who could bias the variability in the results.

With a lack of studies on muscle assessment by ultrasonography in diabetic patients, it is recommended to increase the number of studies that support its use and also to relate the degree of diabetic neuropathy with the characteristics of the ultrasound image.

Regarding OCOMs that measure ulcer-related variables, those valid for size measurement should be validated in future studies for the assessment of ulcer severity. For the diagnosis of osteomyelitis, the probe-to-bone test seems the best alternative to imaging tests (Table 3), although there were no studies on intra- and inter-observer reliability.

The sample selection in terms of diabetes type is important because several OCOMs have been validated only in subjects with a single diabetes type, which, in the case of diabetic neuropathy, is an important factor [67].

### 4.11. Limitations of the Study

Although five languages were introduced in the inclusion criteria for this review, some validated OCOMs could have been excluded in patients with diabetic foot published in a different language; this should be considered before proposing the choice of any of the OCOMs in an absolute manner.

## 5. Conclusion

According to our study, despite the lack of available evidence to define the psychometric properties of the OCOMs, several instruments were found to have enough validity and reliability for clinical use. Diabetic neuropathy assessment via sudomotor analysis, PAD detection by non-invasive electronic devices, wound 3D dimensional measurement, hyperspectral imaging for ulcer prediction and the probe-to-bone test for osteomyelitis diagnosis were highlighted in this study due to the current evidence provided in the available literature.

## Figures and Tables

**Table 1:** Variables related to diabetic neuropathy and the objective clinical outcome measures (OCOMs) validated for their assessment.

Variable	OCOM	Aut	Type	*n*	Sens (%)	Spec (%)	PPV (%)	NPV (%)	Lr+	Lr−	AUC-ROC (%)	Gold Standard (GS)	Agreement with GS	**Inter-Rater**	**Intra-Rater**
Assessment of peripheral neuropathy *	Neuropad	Papanas et al. 2007 [12]	2	120	93 to 100	97 to 100	_	_	_	_	_	MNSI	tau-b = 0.848	_	_
	10-g monofilament	Bracewell et al., 2012 [13]	1/2	141	84	83	78	88	5.01	0.19	_	Neurothesiometer	_	_	_
	Neurotip	Bracewell et al., 2012 [13]	1/2	141	74	83	75	82	4.4	0.31	_	Neurothesiometer	_	_	_
	128 Hz tuning fork	Bracewell et al., 2012 [13]	1/2	141	69	90	81	83	7.16	0.34	_	Neurothesiometer	_	_	_
	VibraTip	Bracewell et al., 2012 [13]	1/2	141	79	82	75	85	4.39	0.25	_	Neurothesiometer	_	_	*r* = 0.88 (*n* =18)
	NeurAp-pathy App	Maliket al., 2011 [14]	1/2	61	80	95	94	83	_	_	_	_	Accuracy = 88%	_	_
	DPN-Check	Shibata et al., 2019 [15]	1/2	57	86.5	43.8	_	_	_	_	0.582; 0.696	Sural nerve conduction velocity (SNCV); Sural nerve conduction amplitude	*r* = 0.81; *r* = 0.62	ICC: 0.807; 0.783	ICC: 0.842; 0.877
	Tactile Circunferencial Discriminator (TDC)	Vileikyte et al., 1997 [16]	1/2	133	92.3	64.2	_	_	_	_	_	Biosthesiometer; S-W Monofilament	*r*^2^ = 0.76; *r*^2^ = 0.73	_	_
	Sudoscan	Jin et al., 2017 [17]	2	60	88.2 to 89.8	41.2 to 46.9	_	_	_	_	0.61 to 0.713	NCS	_	_	_
	Footboard (FB)	Bijli et al., 2017 [18]	_	244	63 to 100	9 to 90	58 to 93	48 to 100	_	_	_	S-W monofilament; 128 Hz tuning-fork		_	_
**Diabetic autonomic neuropathy (DAN)**	Vagus^®^	Ejskjaer et al., 2008 [19]	1	18/323	-	-	-	-	-	-	-	Varia Pulse TF3	*r*^2^ = 0.81–0.98	0.66–0.94	0.85–0.91
Transcutaneous partial pressure of oxygen (TcPO2) *	TCM 400 system	Deng et al., 2014 [20]	2	381	61.1	73.8	_	_	_	_	0.722	NCS	*p* < 0.01	_	_
Current perception threshold *	Neurometer	Masson et al., 1989 [21]	1/2	121	_	_	_	_	_	_	_	Peroneal motor conduction velocity; Biosthesiometer; Thermoesthesiometer	PCC: (−0.66); 0.69; 0.69.	_	_
Assessment of small fiber diabetic neuropathy *	Neuropad	Ponirakis et al., 2014 [22]	1/2	127	68 to 83	49 to 80	26 to 54	44 to 95	1.33 to 4.15	0.21 to 0.65	0.60 to 0.85	MEDOC TSA II; Corneal nerve fiber density and length	_	_	_
	NerveCheck (cold perception part only)	Ponirakis et al., 2016 [23]	_	130	53; 67	82; 85	_	_	_	_	0.7; 0.78	Intradermal epidermic nerve fiber density; Corneal nerve fiber density	_		
Assessment of large fiber diabetic neuropathy *	Neuropad	Ponirakis et al., 2014 [22]	1/2	127	64 to 83	50 to 64	26 to 63	39 to 91	1.39 to 1.94	0.32 to 0.67	0.66 to 0.73	NCS; neurothesiometer; NDS	_	_	_
	NerveCheck (vibration perception part only)	Ponirakis et al., 2016 [23]	_	130	88	82	_	_	_	_	0.84	SNCV			
Assessing nerve conduction *	Electromyograph (EMG)	Shibata et al., 2019 [15]	1/2	57	96.2	40.6	71.4	66.7	_	_	0.615; 0.721	DPN Check	_	ICC: 0.74–0.79	ICC:0.52−0.88
Loss of foot sensation *	Ipswich Touch Test	Sharma et al., 2014 [24]	_	331	78.3 to 81.2	93.9 to 96.4	81.2 to 89.9	92.8 to 96.9	12.9 to 15	0.05 to 0.23	0.87 to 0.97	10-g Neuropen monofilament	_	_	_
	NerveCheck	Ponirakis et al., 2016 [23]	_	130	84	81	_	_	4.36	_	0.72 to 0.86	Neurothesiometer; TSA-II-NeuroSensory Analyser	_	_	0.71–0.86
Vibration perception thresholds *	Neurothesiometer	Bril et al., 1997 [25]	_	152	_	_	_	_	_	_	_	NCS	*r^2^*= 0.228–0.307	_	_
	Vibratron	Bril et al., 1997 [25]	_	152	_	_	_	_	_	_	_	NCS	*r^2^*= 0.042–0.120	_	_
	NerveCheck	Ponirakis et al., 2016 [23]	_	130	88	82	_	_	_	_	0.82 to 0.84	SNCV; sural nerve action potential	_	_	_
Neuropathic pain *	NerveCheck	Ponirakis et al., 2016 [23]	_	130	40 to 70	68 to 84	_	_	_	_	0.7	McGill Pain Questionnaire	_	_	_
Cold perception testing *	NerveCheck	Ponirakis et al., 2016 [23]	_	130	53	82	_	_	_	_	0.7	Intradermal epidermic nerve fiber density	_	_	_
Warm perception testing *	NerveCheck	Ponirakis et al., 2016 [23]	_	130	56	81	_	_	_	_	0.71	Intradermal epidermic nerve fiber density	_	_	_
Atrophy of foot muscles **	Ultrasonography	Severinsen et al., 2007 [26]	1/2	52	_	_	_	_	_	_	_	MRI	*r^2^*=0.71–0.77	_	_
Assessment of autonomic neuropathy ***	Sudoscan	Jin et al., 2017 [17]	2	60	73.9 to 85.6	67.3 to 76.1	_	_	_	_	0.704 to 0.859	NCS	_	_	_
	Neuropad	Spallone et al., 2009 [27]	1/2	51	73 to 82	27 to 75	24 to 44	85 to 91	1.13 to 2.92	0.34 to 0.67	0.71	“Deep breathing. lying to standing. Valsalva and postural hypotension tests”	_	_	_

Authors of the original study (AUT); type of diabetes (TYPE); sensitivity (SENS); specificity (SPEC); positive predictive value (PPV); negative predictive value (NPV); positive likelihood ratio (LR+); negative likelihood ratio (LR−); area under the receiver operator characteristic curve (AUC-ROC); gold standard used for external validity (GOLD STANDARD); degree of external validity with the gold standard (AGREEMENT WITH GS); inter-rater reliability (INTER-RATER); intra-rater reliability (INTRA-RATER). Variable regarding peripheral neuropathy (*); variable regarding proximal neuropathy (**); variable regarding autonomic neuropathy (***).

**Table 2:** Variables related to PAD and the OCOMs validated for their assessment.

Variable	OCOM	Aut	Type	*n*	Sens (%)	Spec (%)	PPV (%)	NPV (%)	Lr+	Lr−	AUC-ROC (%)	Gold standard (GS)	Agreement with GS	Inter-rater	Intra-rater
**Peripheral arterial disease**	Ankle Brachial Index (ABI)	Tehan et al., 2015 [28]	_	117	45.16	92.68	82.35	69.09	6.17	0.59	0.58	Color Duplex Ultrasound	_	_	ICC = 0.62
	Toe Brachial Index (TBI)	Tehan et al., 2015 [28]	_	117	63.64	82.05	75	72.73	10.39	0.28	0.75	Color Duplex Ultrasound	_	_	ICC = 0.8
**Transcutaneous partial pressure of oxygen (TcPO2)**	Novametrix 800 monitor	Ballard et al., 1995 [29]	1/2	55	98	44	91	80	_	_	_	_	Accuracy: 90%	_	_
**Measurement of ABI**	OMRON BP-203RPEIII	Ma et al., 2017 [30]	_	230	94.5	98.99	_	_	55.12	0.056	0.981	Eco-Doppler	K = 0.928	_	_

Authors of the original study (AUT); type of diabetes (TYPE); sensitivity (SENS); specificity (SPEC); positive predictive value (PPV); negative predictive value (NPV); positive likelihood ratio (LR+); negative likelihood ratio (LR−); area under the receiver operator characteristic curve (AUC-ROC); gold standard used for external validity (GOLD STANDARD); degree of external validity with the gold standard (AGREEMENT WITH GS); inter-rater reliability (INTER-RATER); intra-rater reliability (INTRA-RATER).

**Table 3:** Variables related to ulcer characteristics and the OCOMs validated for their assessment; sensitivity (SENS).

VARIABLE	OCOM	AUT	TYPE	*n*	SENS (%)	SPEC (%)	PPV (%)	NPV (%)	LR+	LR−	AUC-ROC (%)	GOLD STANDARD (GS)	AGREEMENT WITH GS	INTER-RATER	INTRA-RATER
**Wound area measurement**	ImageJ	Aragón-Sánchez et al., 2017 [31]	_	25	_	_	_	_	_	_	_	_	_	ICC = 1	ICC = 0.99
	SilhouetteMobile	Foltynski et al., 2013 [32]	_	16	_	_	_	_	_	_	_	Elliptical method	MAE = 1.7 to 4.5	_	_
	VisiTrak	Foltynski et al., 2013 [32]	_	16	_	_	_	_	_	_	_	Elliptical method	MAE = 1.8 to 3	_	_
	TeleDiaFos	Foltynski et al., 2013 [32]	_	16	_	_	_	_	_	_	_	Elliptical method	MAE = 1.7 to 12.9	_	_
**Wound area and volume measurement**	3D Wound Assessment Camera	Jorgensen et al., 2018 [33]	_	47	_	_	_	_	_	_	_	3D camera; gel injection	ICC = 0.975 ICC = 0.977	ICC = 0.946 to 0.999	ICC = 0.971 to 0.997
**Assessment of foot infection**	Photographic Foot Imaging Device (PFID)	Hazenberg et al., 2014 [34]	_	38	57	86	73	76	_	_	_	Live assessment	_	ICC = 0.44	ICC = 0.52 to 0.77
**Diagnosis of ulcer**	PFID	Hazenberg et al., 2010 [35]	_	32	88	98	_	_	_	_	_	Live assessment	Kappa = 0.87	ICC = 0.74 to 0.88	ICC = 0.91 to 1
**Diagnosis of callus**	PFID	Hazenberg et al., 2010 [35]	_	32	69	89	_	_	_	_	_	Live assessment	Kappa = 0.61	ICC = 0.52 to 0.73	ICC = 0.7 to 1
**Diagnosis of absence of signs**	PFID	Hazenberg et al., 2010 [35]	_	32	90	90	_	_	_	_	_	Live assessment	Kappa = 0.83	ICC = 0.62 to 0.73	ICC = 0.89 to 1
**Severity of diabetic foot ulcer**	Plasma fibrinogen via immunoturbidimetric assay	Li et al., 2014 [36]	_	152	80.9	82.6	78.6	89	_	_	0.858	Neutrophil counting; white blood cell counting, C-reactive protein	SCC = 0.614; 0.616; 0.705	_	_
**Predicting risk of ulcer formation**	Hyperspectral imaging device	Yudovsky et al., 2011 [37]	1/2	66	0 to 100	72 to 100	_	_	_	_	0.89	_	_	_	_
**Diagnosis of osteomielitis**	Probe-to-bone test	Morales-Lozano et al., 2016 [38]	1/2	132	98.1	77.78	94.5	91.3	4.45	0.02	_	Intraoperative histology and culture	Kappa = 0.803	_	_
	Plain radiography	Nawaz et al. 2009 [39]	_	110	63	87	60	88	_	_	_	Intraoperative histology and culture	Accuracy = 81%	_	_
	[18F]-2-fluoro-2-deoxy-Dglucose (FDG)-positron emission tomography (PET)	Nawaz et al. 2009 [39]	_	110	81	93	78	94	_	_	_	Intraoperative histology and culture	Accuracy = 90%	_	_
	MRI	Nawaz et al. 2009 [39]	_	110	91	78	96	57	_	_	_	Intraoperative histology and culture	Accuracy = 81%	_	_
	Leucocyte counting	Ertugrul et al., 2006 [40]	_	31	91	67	95	50	_	_	_	CT scan contrast with Tc99	_	_	_

Authors of the original study (AUT); type of diabetes (TYPE); sensitivity (SENS); specificity (SPEC); positive predictive value (PPV); negative predictive value (NPV); positive likelihood ratio (LR+); negative likelihood ratio (LR−); area under the receiver operator characteristic curve (AUC-ROC); gold standard used for external validity (GOLD STANDARD); degree of external validity with the gold standard (AGREEMENT WITH GS); inter-rater reliability (INTER-RATER); intra-rater reliability (INTRA-RATER).
